# miR-26a/30d/152 are reliable reference genes for miRNA quantification in skin wound age estimation

**DOI:** 10.1093/fsr/owad037

**Published:** 2023-10-15

**Authors:** Longlong Suo, Jian Cheng, Haomiao Yuan, Zhenfei Jiang, Dilichati Tash, Linlin Wang, Hao Cheng, Zhongduo Zhang, Fuyuan Zhang, Miao Zhang, Zhipeng Cao, Rui Zhao, Dawei Guan

**Affiliations:** Department of Forensic Pathology, China Medical University School of Forensic Medicine, Shenyang, China; Department of Forensic Pathology, China Medical University School of Forensic Medicine, Shenyang, China; Department of Forensic Pathology, China Medical University School of Forensic Medicine, Shenyang, China; Department of Road Traffic Accident Investigation, Academy of Forensic Science, Shanghai, China; Department of Forensic Pathology, China Medical University School of Forensic Medicine, Shenyang, China; Autonomous Prefecture Public Security Bureau, Xinjiang Uygur Autonomous Region, Urumqi, China; Department of Forensic Pathology, China Medical University School of Forensic Medicine, Shenyang, China; Collaborative Laboratory of Intelligentized Forensic Science, Shenyang, China; Laboratory of Forensic Biochemistry, China Medical University School of Forensic Medicine, Shenyang, China; Department of Forensic Pathology, China Medical University School of Forensic Medicine, Shenyang, China; Department of Forensic Pathology, China Medical University School of Forensic Medicine, Shenyang, China; Department of Forensic Pathology, China Medical University School of Forensic Medicine, Shenyang, China; Department of Forensic Pathology, China Medical University School of Forensic Medicine, Shenyang, China; Collaborative Laboratory of Intelligentized Forensic Science, Shenyang, China; Laboratory of Forensic Biochemistry, China Medical University School of Forensic Medicine, Shenyang, China; Department of Forensic Pathology, China Medical University School of Forensic Medicine, Shenyang, China; Collaborative Laboratory of Intelligentized Forensic Science, Shenyang, China; Laboratory of Forensic Biochemistry, China Medical University School of Forensic Medicine, Shenyang, China; Department of Forensic Pathology, China Medical University School of Forensic Medicine, Shenyang, China; Collaborative Laboratory of Intelligentized Forensic Science, Shenyang, China; Laboratory of Forensic Biochemistry, China Medical University School of Forensic Medicine, Shenyang, China; Department of Forensic Pathology, China Medical University School of Forensic Medicine, Shenyang, China; Collaborative Laboratory of Intelligentized Forensic Science, Shenyang, China; Laboratory of Forensic Biochemistry, China Medical University School of Forensic Medicine, Shenyang, China

**Keywords:** forensic sciences, forensic genetics, reference gene, miRNA, wound age estimation, skin wound healing, normalization, qRT-PCR

## Abstract

MicroRNAs (miRNAs) are a class of small non-coding RNAs that exert their biological functions as negative regulators of gene expression. They are involved in the skin wound healing process with a dynamic expression pattern and can therefore potentially serve as biomarkers for skin wound age estimation. However, no reports have described any miRNAs as suitable reference genes (RGs) for miRNA quantification in wounded skin or samples with post-mortem changes. Here, we aimed to identify specific miRNAs as RGs for miRNA quantification to support further studies of skin wound age estimation. Overall, nine miRNAs stably expressed in mouse skin at certain posttraumatic intervals (PTIs) were preselected by next-generation sequencing as candidate RGs. These nine miRNAs and the commonly used reference genes (comRGs: U6, GAPDH, ACTB, 18S, 5S, LC-Ogdh) were quantitatively examined using quantitative real-time reverse-transcription polymerase chain reaction at different PTIs during skin wound healing in mice. The stabilities of these genes were evaluated using four independent algorithms: GeNorm, NormFinder, BestKeeper, and comparative Delta Ct. Stability was further evaluated in mice with different post-mortem intervals (PMIs). Overall, mmu-miR-26a-5p, mmu-miR-30d-5p, and mmu-miR-152-3p were identified as the most stable genes at both different PTIs and PMIs. These three miRNA RGs were additionally validated and compared with the comRGs in human samples. After assessing using one, two, or three miRNAs in combination for stability at different PTIs, PMIs, or in human samples, the set of miR-26a/30d/152 was approved as the best normalizer. In conclusion, our data suggest that the combination of miR-26a/30d/152 is recommended as the normalization strategy for miRNA qRT-PCR quantification in skin wound age estimation.

**Key points:**

- The small size of miRNAs makes them less susceptible to post-mortem autolysis or putrefaction, leading to their potential use in wound age estimation.
- Studying miRNAs as biological indicators of skin wound age estimation requires the selection and validation of stable reference genes because commonly used reference genes, such as U6, ACTB, GAPDH, 5S, 18S, and LC-Ogdh, are not stable.
- miR-26a/30d/152 are stable and reliable as reference genes and their use in combination is a recommended normalization strategy for miRNA quantitative analysis in wounded skin.

## Introduction

Elucidating the sequential histopathological and molecular biological changes that occur during the skin wound healing process can help clarify the specific mechanism involved and determine the age of the wounds. In general, the protein or mRNA levels of the chemokines and cytokines that are dynamically expressed during skin wound healing may be detected and evaluated by biological techniques such as Western blotting or quantitative polymerase chain reaction (qPCR), but these methods cannot help interpret wound age [1–3]. However, biomarker determination is often influenced by post-mortem autolysis or putrefaction of the tissues examined, which might interfere with wound age estimation in forensic practice. Therefore, it is necessary to identify certain biological indicators that are relatively stable and less affected by such factors.

MicroRNAs (miRNAs) are a class of endogenous single-stranded non-coding RNAs (18–24 nucleotides) that are widely expressed in eukaryotes and viruses [4]. By complementary binding to the 3′ untranslated region (3′ UTR) of target gene mRNAs, they can promote the degradation or inhibit the translation of these mRNAs at the post-transcriptional level [4–6]. Currently, many studies have revealed that miRNAs exert their biological functions in skin wound healing with a dynamic expression pattern: in keratinocytes, miR-132 decreased the production of chemokines and the capability to attract leukocytes by suppressing the NF-κB pathway; miR-31 mediates inflammatory signaling to promote re-epithelialization during skin wound healing [7–9]. In addition, the short size of miRNAs makes them less susceptible to degradation caused by chemicals and post-mortem autolysis or putrefaction. With these biological functions and characteristics, miRNAs could potentially be applied to the identification of body fluids or tissues [10–12], estimation of the post-mortem interval (PMI) [13–15], or cause-of-death assessment [16–18], which are all significant in the forensic science field. Therefore, we hypothesize that miRNAs can serve as a bio-indicator that is potentially applicable to skin wound age estimation. However, it is imperative to determine and select the optimal reference genes (RGs) before experimentally testing this hypothesis.

Currently, the qPCR assay is a gold standard in the field and is most commonly used for quantitative analysis of miRNAs. Effective RGs are indispensable for data normalization and not affected by the potential technical bias introduced during each step of reverse transcription and quantification during qPCR [19, 20]. U6 is currently the most commonly used RG for miRNA expression normalization, but remains controversial because of increasing evidence of its unstable expression in serum, heart, liver and its post-mortem degradation [13, 21]. Using an RG as an internal control is the most frequently used method for miRNA data normalization. However, an appropriate RG for this purpose has not been identified for qPCR-based miRNA quantification in wounded skin samples or those with post-mortem changes. In the present study, we primarily preselected specific miRNAs as potential RG candidates in mouse wounded skin tissues by next-generation sequencing (NGS). We evaluated their stability during skin wound healing and at different PMIs to confirm these miRNAs as candidate RGs for the subsequent investigation of skin wound age estimation.

## Materials and methods

### Animal model of incised skin wound and sample collection

The establishment of an incised skin wound animal model was previously described [22]. Briefly, healthy male C57BL/6J mice (7–8 weeks old) weighing 22 ± 2 g were used. After hair removal and sterilization, the mice were anesthetized with isoflurane and a full-thickness 1.5-cm-long skin incision was created on the central dorsum. Mice without incised wounds served as a normal control (NC). Each mouse was individually housed in a temperature-controlled animal room with a 12-h light/dark cycle under specific pathogen-free conditions. The animals had access to commercial mouse chow and distilled water *ad libitum*.

For small RNA sequencing and miRNA screening, mice with cutaneous wounds were sacrificed at 12 h, 3 days, 7 days, or 14 days post-injury (three mice at each time point). Another three mice without wounds were used as the NC. For validation of miRNAs and evaluation of their stability at different posttraumatic intervals (PTIs), 50 mice with cutaneous wounds were sacrificed at 12 h, 1 day, 3 days, 5 days, 7 days, 9 days, 11 days, 13 days, 17 days, or 21 days post-injury (five mice at each time point). Another five mice without injury were used as the NC. For assessment of miRNA stability at different PMIs, another 30 mice with cutaneous wounds were sacrificed at 5 days post-injury and randomly divided into six post-mortem groups: PMI = 0, 1.5, 3, 4.5, 6, or 7.5 days (five mice at each time point). The animals were housed in a controlled environment incubator at an ambient temperature of (25 ± 2) °C and humidity of (55 ± 3)%. All mice were sacrificed by carbon dioxide euthanasia. A 2.0 × 1.0-cm skin sample was taken from each wounded and NC mouse. All samples were snap frozen in liquid nitrogen and stored at −80 °C before RNA extraction. The animal experiments were performed in accordance with the Guide for the Care and Use of Laboratory Animals and approved by the Animal Experiment Committee of China Medical University (cmu2019119).

### Human skin sample collection

For validation of miRNA RGs in the human skin, samples were obtained from the dermal tissue of stab wounds, surgical wounds, and lacerations within 1 cm of the incisions of 15 individuals who died from trauma and were autopsied at China Medical University School of Forensic Medicine. The dermal tissue collected at the contralateral site was used as a control. Samples were frozen immediately after sampling and stored at −80 °C in TRIzol solution (Invitrogen, Life Technologies, Waltham, MA, USA). The survival period after wounding, site, cause of death, and intervals between death and autopsy are listed in Table 1. Autopsy records and pathology reports confirmed all details. Written informed consent was obtained from relatives of the deceased in all cases, and protocols were approved by the Scientific and Ethical Committee of China Medical University (ethical review [2018] 062) and performed in accordance with the 1964 Declaration of Helsinki and its later amendments.

**Table 1:** Human sample information.

Ind no	Gender	Age (year)	SPAW	Site	Injury tape	PMI (day)	T ( °C)	Causes of death
1	M	28	Instantaneous	Upper limb	Stab wound	2	RT	Traumatic shock
2	F	54	Instantaneous	Foot	Laceration	8	−20 to −18	Traumatic shock
3	M	72	Instantaneous	Back of hand	Laceration	14	−20 to −18	Traumatic shock
4	F	34	Instantaneous	Temporal	Laceration	7	−20 to −18	Traumatic shock
5	F	63	4 h	Parietal region	Laceration	10	−20 to −18	Traumatic shock
6	M	57	4.5 h	Wrist	Laceration	23	−20 to −18	Traumatic shock
7	M	65	21 h	Back	Stab wound	2	RT	Haemorrhagic shock
8	M	70	3.5 d	Parietal region	Laceration	22	−20 to −18	Diffuse axonal injury
9	M	82	3–4 d	Anterior tibial	Laceration	10	−20 to −18	Crush syndrome
10	F	83	5 d	Foot	Surgical wound	21	−20 to −18	Traumatic shock
11	M	77	15 d	Abdomen	Surgical wound	23	−20 to −18	Peritonitis
12	M	72	16 d	Foot	Laceration	16	−20 to −18	Cerebral haemorrhage
13	F	86	19 d	Foot	Laceration	16	−20 to −18	Cerebral infarction
14	M	55	22 d	Anterior tibial	Laceration	28	−20 to −18	Traumatic shock
15	M	67	23 d	Temporal	Surgical wound	9	−20 to −18	Craniocerebral injury

Ind no: number of individuals; SPAW: survival period after wounding; PMI: post-mortem interval (between death and autopsy); d: days; RT: room temperature.

### Preselection of miRNA candidate RGs by NGS

Total RNA from animal and human samples was isolated with TRIzol (Invitrogen) reagent according to the manufacturer’s protocol. RNA samples were normalized to 500 ng/μL according to the recommended concentration range of the kit. The cDNA library was obtained by reverse transcription and enrichment of the 140–160-bp-size PCR products. Small RNA sequencing was conducted with the Illumina HiSeq 2500 platform (Guangzhou Gene Denovo Co. Ltd, Guangzhou, China). The miRNA expression level was normalized to transcripts per million (TPM) with the formula: TPM = (Actual miRNA counts/Total counts of clean tags) × 10^6^.

To comprehensively preselect the miRNAs with stable and non-differential expression, the coefficient of variation (CV) was calculated using the miRNA actual count value (non-normalized) and TPM value (normalized), respectively. The miRNAs with both the lowest CV and higher expression levels were selected as the miRNA candidate RGs. The higher expression abundance was determined by count value >8 000 or TPM > 5 000.

### Validation of miRNA candidate RGs and detection of commonly used RGs by qRT-PCR

Next, qRT-PCR was performed using a Roche LightCycler 480 fluorescence quantitative PCR instrument (Roche, Basel, Switzerland). For verification of miRNA candidate RGs, miRNA reverse-transcription reactions and amplification were performed using the TransScript First-Strand cDNA Synthesis SuperMix and Tip Green qPCR SuperMix (TransGen Biotech, Beijing, China).

For comparison of the stability of miRNA candidate RGs with commonly used RGs (comRGs), including U6, ACTB, GAPDH, 5S, 18S, and LC-Ogdh, quantitative detection of the comRGs was conducted by reverse-transcription reactions and amplification using the PrimeScript RT-PCR kit and TB Green Premix Ex Taq II (TaKara, Shiga, Japan).

All samples were run in technical duplicates, and the mean Ct value of each gene was used in the analysis. The primer sequences are listed in Supplementary Table S1.

### Data analysis and statistics

To compare gene expression stability and rank, four different statistical algorithms, including GeNorm, NormFinder, BestKeeper, and the comparative Delta Ct, were used to evaluate the stability of miRNAs as candidate RGs and the comRGs mentioned above. GeNorm evaluates the most stable RGs by gene stability measure (*M* value) and provides a reference for the optimal number of RGs required for standardization [23]. NormFinder is based on a mathematical model that ranks genes by calculating the “stability value” of candidates, which is estimated by the intra- and inter-group variation values [24]. BestKeeper determines the stability of candidate genes based on the standard deviation (SD) and pairwise correlation analysis of their Ct values [25]. The comparative Delta Ct method compares the relative expression of gene pairs within each sample and ranks the stability of RGs according to the repeatability of the gene expression difference [26]. The geometric mean (geomean) of each RG ranking across the four programmes was eventually determined, leading to a consensus stability score.

## Results

### Preselection of miRNA candidate RGs from NGS data

**Figure 1:**
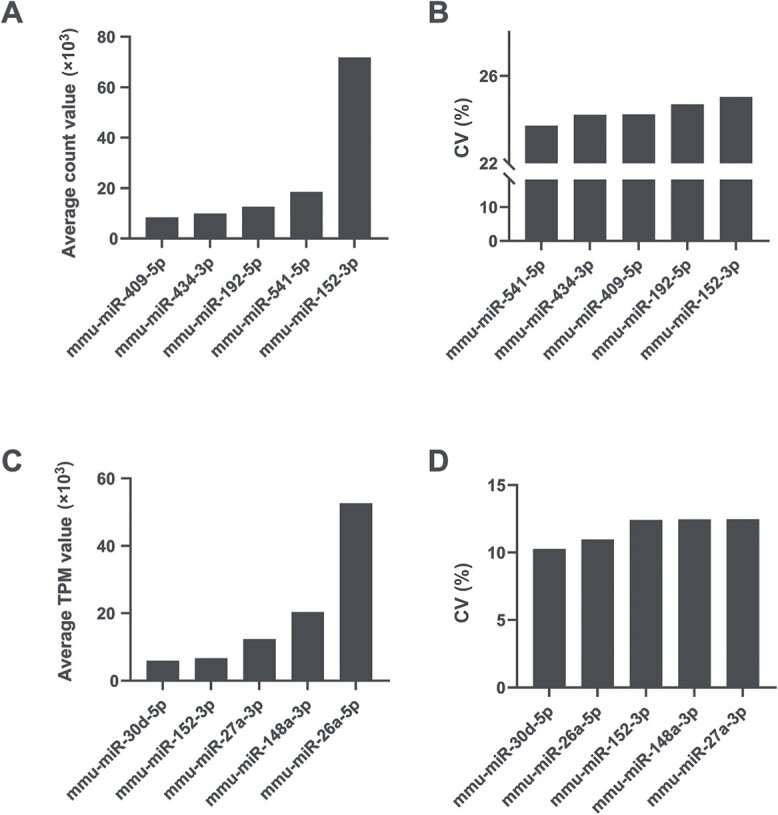
The five miRNAs with the lowest coefficient of variation (CV) values and higher abundance preselected by the count value or transcripts per million (TPM) value of miRNA from the next-generation sequencing (NGS) data. (A) The miRNAs preselected by raw count values. (B) The CV of miRNAs preselected by raw count values. (C) The miRNAs preselected by TPM values. (D) The CV of miRNAs preselected by TPM values.

To comprehensively select the miRNAs with stable and non-differential expression during skin wound healing, miRNAs were preselected by NGS in normal skin and injured skin samples taken at 12 h, 3 days, 7 days, and 14 days post-injury. The miRNA expression profile was obtained from the NGS data. A total of 563 different miRNAs were detected after quality control. The CV was calculated by raw count value (non-normalized) or TPM value (normalized) of the miRNAs at each time point post-injury. For calculation methods, CV = (SD/Mean) × 100%. Five miRNAs with both the lowest CV calculated by count value and higher expression abundance and five miRNAs with both the lowest CV calculated by TPM value and higher expression abundance were preselected as the RG candidates (Figure 1).

The five miRNAs with the lowest CV calculated by count value and higher abundance were mmu-miR-541-5p, mmu-miR-434-3p, mmu-miR-409-5p, mmu-miR-192-5p, and mmu-miR-152-3p. Another five miRNAs with the lowest CV calculated by TPM value and higher abundance were mmu-miR-30d-5p, mmu-miR-26a-5p, mmu-miR-148a-3p, mmu-miR-27a-3p, and mmu-miR-152-3p. Because mmu-miR-152-3p was preselected with both the count value and TPM, a total of nine miRNAs were recruited as candidate RGs for further evaluation in subsequent studies. None of the preselected candidates resided within the same gene cluster, which therefore reduced the likelihood of including co-regulated miRNAs in the stability analysis [27, 28].

### Evaluation of the stability of the preselected miRNA candidate RGs compared with that of the comRGs during skin wound healing in mice

#### Expression levels of miRNA candidate RGs and comRGs

To visualize the expression levels and variation of the preselected miRNA candidate RGs and comRGs (U6, ACTB, GAPDH, 5S, 18S, and LC-Ogdh), the raw Ct values were acquired using qRT-PCR assays in mouse skin samples taken at 12 h, 1 day, 3 days, 5 days, 7 days, 9 days, 11 days, 13 days, 17 days, and 21 days post-wounding.

**Figure 2:**
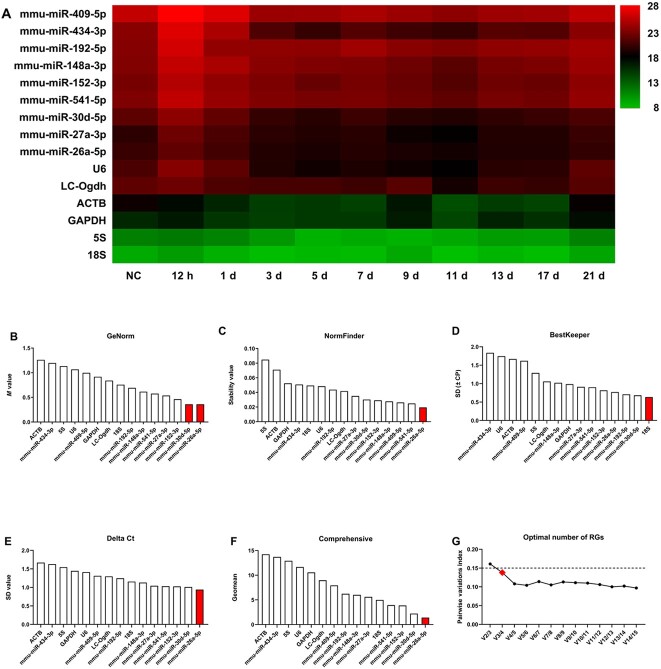
The raw Ct values and stability analysis of miRNA candidate reference genes (RGs) and commonly used RGs (comRGs) during skin wound healing. (A) Heatmap of raw Ct values of miRNA candidate RGs and comRGs during skin wound healing. The stability of miRNA candidate RGs and comRGs during skin wound healing were analyzed using the (B) GeNorm, (C) NormFinder, (D) BestKeeper, and (E) comparative Delta Ct algorithms. (F) The comprehensive ranking was obtained from the geometric mean of each algorithm’s ranking. (G) Pairwise variation analysis showing the optimal number of RGs for normalization.

The mean Ct value for each RG was calculated with the Ct values obtained at different PTIs. As shown in Figure 2A (Supplementary Table S2), the 15 genes (nine miRNA candidate RGs and six comRGs) showed mean Ct values across a wide spectrum, ranging from 8.60 ± 0.75 (18S rRNA) to 25.77 ± 1.88 (mmu-miR-409-5p). The expression levels of the nine miRNAs validated by qRT-PCR were similar to the corresponding NGS data (Supplementary Figure 1 and Supplementary Table S3). In addition, the expression patterns of the nine miRNAs were stable at different PTIs in both the NGS and qRT-PCR data sets. Notably, mmu-miR-26a-5p was expressed with the highest abundance (Ct value = 19.41 ± 1.03), followed by mmu-miR-27a-3p (Ct value = 20.04 ± 1.22) and mmu-miR-30d-5p (Ct value = 20.76 ± 0.88), whilst mmu-miR-409-5p had the lowest abundance (Ct value = 25.77 ± 1.88). The comRG 18S rRNA showed the most abundant expression (Ct value = 8.60 ± 0.75), followed by 5S rRNA (Ct value = 10.01 ± 1.52) and GAPDH (Ct value = 15.87 ± 1.26), whilst U6 was the lowest comRG in abundance (Ct value = 20.70 ± 2.02).

#### Stability analysis of miRNA candidate RGs and comRGs

To compare gene expression stability and rank, four different statistical algorithms, including GeNorm, NormFinder, BestKeeper, and the comparative Delta Ct, were used to evaluate the stability of miRNA candidate RGs in comparison with the comRGs. As shown in Figure 2B–G (Supplementary Table S4), different stability and rank values were obtained with the four algorithms.

*GeNorm.* According to the GeNorm algorithm, all 15 genes showed an *M* value lower than 1.5 (the *M* value set by default as the threshold to identify normalizers). Therefore, all the genes evaluated could potentially be utilized as RGs. However, GeNorm identified mmu-miR-26a-5p and mmu-miR-30d-5p as the most stable RGs with the lowest *M* value of 0.361, followed by mmu-miR-152-3p (*M* value = 0.462) (Figure 2B). ACTB (*M* value = 1.260) was determined to be the least stable miRNA amongst the 15 genes. Furthermore, GeNorm also suggested the optimal number of RGs determined by calculating pairwise variation values. As shown in Figure 2G, V3/4 showed a *V* value of 0.138 below the threshold of 0.15, indicating that a minimum of three genes were necessary for an adequate normalization amongst the 15 genes.

**Figure 3:**
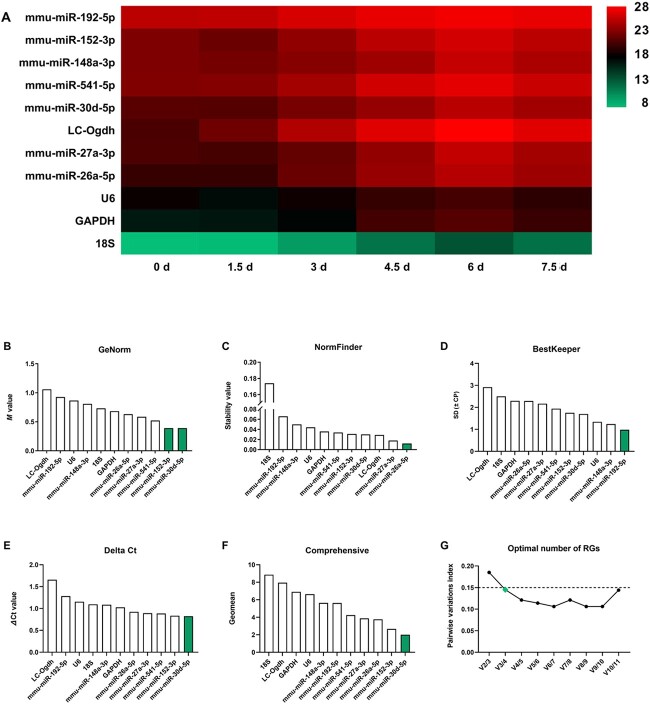
The Ct values and stability analysis of miRNA candidate reference genes (RGs) and commonly used RGs (comRGs) at different post-mortem intervals (PMIs). (A) Heatmap of the raw Ct values of miRNA candidate RGs and comRGs at different PMIs. The stability of miRNA candidate RGs and comRGs at different PMIs were analyzed using (B) GeNorm, (C) NormFinder, (D) BestKeeper, and (E) comparative Delta Ct. (F) The comprehensive ranking was obtained from the geometric mean of each algorithm’s ranking. (G) Pairwise variation analysis showing the optimal number of RGs for normalization.

*NormFinder*. NormFinder recommends choosing the candidate RGs with the lowest stability values. Similar to the GeNorm algorithm, NormFinder suggested that mmu-miR-26a-5p was the most stable RG with the lowest stability value of 0.019, followed by mmu-miR-541-5p (stability value = 0.024) and mmu-miR-409-5p (stability value = 0.026). The gene 5S rRNA (stability value = 0.085) was ranked as the least stable gene (Figure 2C).

*BestKeeper*. Using BestKeeper, the most stable gene with the least overall variation was 18S rRNA (SD = 0.635), followed by mmu-miR-30d-5p (SD = 0.682) and mmu-miR-192-5p (SD = 0.709). The least stable gene was mmu-miR-434-3p (SD = 1.835) (Figure 2D).

*The comparative Delta Ct*. The comparative Delta Ct method demonstrated that the three most stable genes were mmu-miR-26a-5p (mean SD = 0.941), mmu-miR-30d-5p (mean SD = 1.011), and mmu-miR-152-3p (mean SD = 1.026), whilst the least stable gene was ACTB (mean SD = 1.671) (Figure 2E).

To integrate and standardize the different rankings obtained using the four algorithms, each gene was ranked according to the four algorithms from rank 1 (most stable) to 15 (least stable). The geometric mean of each RG weight was calculated with the four algorithms and the RG with the lowest value was considered to be the most stable one. Using this approach, mmu-miR-26a-5p, mmu-miR-30d-5p, and mmu-miR-152-3p were the most stable genes, which were therefore identified as the potential RGs. However, ACTB, mmu-miR-434-3p, and 5S were the least stable genes amongst the group and should be precluded as RGs in this study associated with skin wound healing (Figure 2F).

### Evaluation of the stability of the preselected miRNA candidate RGs compared with that of the comRGs in mouse skin at different PMIs

#### Expression levels of miRNA candidate RGs and comRGs

To further evaluate the stability of the miRNA candidate RGs and comRGs at different PMIs, qRT-PCR assays were performed with the injured skin samples taken at 0, 1.5, 3, 4.5, 6, and 7.5 days post-mortem in mice. Generally, the response to the injury is relatively intensive at 5 days post-wounding because the skin is in the active inflammatory phase and proliferative phase of communication at that time [9, 29–31]. The skin samples collected at 5 days post-injury were used to evaluate the stability of the miRNA candidate RGs and comRGs at different PMIs. Because ACTB, mmu-miR-434-3p, 5S, and mmu-miR-409-5p were ranked the least stable in our study and were excluded from further investigation, the remaining 11 genes were recruited for the subsequent analysis of the stability at different PMIs.

The mean Ct value for each gene was calculated with the Ct value obtained at the different PMIs. As shown in Figure 3A (Supplementary Table S5), the Ct values of the 11 genes (seven miRNA candidate RGs and four comRGs) varied widely with PMI extension. The Ct values of LC-Ogdh, mmu-miR-26a-5p, mmu-miR-30d-5p, and mmu-miR-192-5p were significantly increased at 3 days post-mortem, whilst the other genes showed a significant increase at 4.5 days post-mortem.

#### Stability analysis of miRNA candidate RGs and comRGs

The stability of the miRNA candidate RGs and comRGs over different PMIs was calculated using the four algorithms mentioned above (Figure 3B–G and Supplementary Table S6).

*GeNorm.* According to GeNorm, mmu-miR-30d-5p and mmu-miR-152-3p were identified as the most stable pair of miRNAs because of the lowest *M* value (*M* value = 0.391), followed by mmu-miR-541-5p (*M* value = 0.522). LC-Ogdh was considered the least stable gene (*M* value = 1.060) (Figure 3B). Furthermore, GeNorm recommended that at least three genes were required for the optimal number of RGs for normalization because V3/4 showed a *V* value of 0.145 (Figure 3G).

*NormFinder.* Using NormFinder, mmu-miR-26a-5p was suggested as the best RG (stability value = 0.012), followed by mmu-miR-27a-3p (stability value = 0.018) and LC-Ogdh (stability value = 0.029). 18S rRNA was found to be the worst RG (stability value = 0.174) (Figure 3C).

*BestKeeper.* With BestKeeper, mmu-miR-192-5p had the lowest SD (SD = 0.98) and was ranked as the most reliable RG, followed by mmu-miR-148a-3p (SD = 1.24) and U6 (SD = 1.34). LC-Ogdh was considered the least reliable gene (SD = 2.92) (Figure 3D).

*The comparative Delta Ct.* Here, mmu-miR-30d-5p (mean SD = 0.822), mmu-miR-152-3p (mean SD = 0.834), and mmu-miR-541-5p (mean SD = 0.886) were the most stable genes using the comparative Delta Ct method. Like the analyses mentioned above, the results suggested that LC-Ogdh was the poorest candidate (mean SD = 1.658) (Figure 3E).

Using the rankings generated by the four algorithms, a comprehensive ranking was obtained by calculating the geometric mean of each RG weight. Overall, mmu-miR-30d-5p, mmu-miR-152-3p, and mmu-miR-26a-5p were demonstrated to be the most stable genes and were possibly the best RGs. Conversely, 18S rRNA, LC-Ogdh, and GAPDH ranked last and were not suitable as RGs for skin samples with post-mortem changes in this study (Figure 3F).

### Validation stability of miRNA candidate RGs and comRGs in human samples

**Figure 4:**
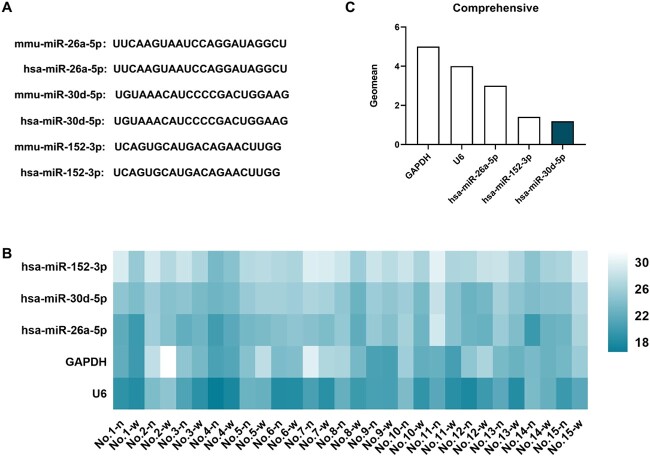
Validation of the stabilities of five genes in human samples. (A) The sequences of miRNAs in *Mus musculus* (mouse) and their corresponding miRNAs in *Homo sapiens* (human) from the miRBase database. (B) The Ct values of miRNA candidate reference genes (RGs) and commonly used RGs (comRGs) in human samples. No.1-n represents Ind No 1 normal skin and No.1-w represents Ind No 1 wounded skin. (C) The comprehensive ranking was obtained from the geometric mean of each algorithm’s ranking.

From the animal experiments mentioned above, mmu-miR-26a-5p, mmu-miR-30d-5p, and mmu-miR-152-3p were identified as the most stable genes evaluated by the stability analysis at both different PTIs and PMIs. According to the high conservation of miRNAs and miRBase database (www.mirbase.org), the sequences of these three miRNAs were completely identical in *Homo sapiens* and *Mus musculus* (Figure 4A). Therefore, we speculated that these three miRNAs could also be used as RGs in human skin samples.

To confirm our speculation, hsa-miR-26a-5p, hsa-miR-30d-5p, and hsa-miR-152-3p as prospective RGs in humans were validated by analyzing 15 normal and 15 wounded human skin samples. The stability of U6 and GAPDH was also compared. The Ct values of the five genes (three miRNAs and two comRGs) showed wide variation, with a range from 20.37 ± 2.11 (U6) to 27.37 ± 1.63 (hsa-miR-152-3p) amongst the 30 skin samples. However, U6 (20.37 ± 2.11) and GAPDH (24.12 ± 2.88) clearly showed high expression and variance across the human samples (Figure 4B and Supplementary Table S7).

The stability of the miRNAs and comRGs in human samples was measured using the four algorithms mentioned previously. Finally, the comprehensive ranking of the stability analysis of the six genes in human samples was obtained. The three most stable genes were hsa-miR-30d-5p, hsa-miR-152-3p, and hsa-miR-26a-5p, whilst the least stable gene was GAPDH (Figure 4C and Supplementary Table S8).

### Adequate normalization strategy for utilization of the three miRNAs as RGs

**Figure 5:**
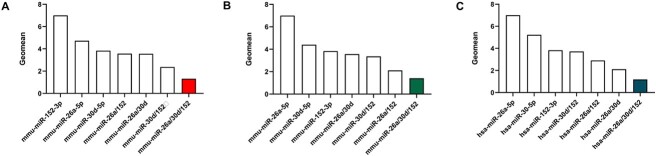
Assessment of using multiple miRNAs as reference genes (RGs) for reliable normalization. (A) Data at different posttraumatic intervals in mice. (B) Data at different post-mortem intervals in mice. (C) Data at different posttraumatic intervals in humans.

From the comprehensive evaluation mentioned above, mmu/hsa-miR-26a-5p, mmu/hsa-miR-30d-5p, and mmu/hsa-miR-152-3p were chosen as the miRNA RGs. To assess the stability of the miRNA RGs, the stabilities of single, two, or three miRNA combinations (geometric mean of Ct values) were further verified at different PTIs, PMIs, or human samples using the four algorithms mentioned above. As shown in Figure 5 (Supplementary Tables S9–11), the two- or three-miRNA combination was better than a single miRNA in stability for an endogenous control gene. Overall, a set of three miRNAs (miR-26a/30d/152) was recommended as the best normalization strategy amongst all of our experimental conditions. This combination was first in the rankings, as evaluated with the four algorithms.

## Discussion

The estimation of wound age remains an unresolved issue in the forensic science field. Until now, researchers have proposed many biomarkers such as cytokines [interleukine-1 (IL-1), tumour necrosis factor (TNF)], chemokines [IL-8, macrophage chemotactic protein-1 (MCP-1)], growth factors [heparin-binding epidermal growth factor (EGF), fibroblast growth factor (FGF)] that could be applied to skin wound age estimation [32, 33]. Interestingly, miRNAs have been reported to actively participate in skin wound repair [7–9] and are believed to be more applicable to wound age estimation because of their specific biological characteristics such as less susceptible to degradation caused by chemicals and postmortem autolysis or putrefaction [11]. In recent years, further research on miRNAs has led to an increasing number of researchers proposing that using small nuclear RNAs (snRNAs), such as U6 and U6B, as internal controls for miRNA quantification experiments has critical pitfalls. The snRNA and miRNA biogenesis processes are separate. Specifically, snRNAs are not processed by the spliceosome, but by the Drosha complex, and do not have similar physicochemical properties to miRNA molecules [34]. Moreover, different RNA classes may vary from miRNAs in terms of efficiency in the RNA isolation, cDNA synthesis, and amplification processes [35]. Therefore, selecting the same RNA class for miRNA quantification normalization may be the most scientifically appropriate approach [35–37].

At present, few studies have reported on the selection of miRNA RGs for qRT-PCR experiments in work associated with skin wound repair. In our study, we noted that the stabilities of the preselected miRNA candidates were better than those of the comRGs by comprehensive analyses with the GeNorm, NormFinder, BestKeeper, and comparative Delta Ct algorithms. These analyses suggested that ACTB was less stable relative to the specific miRNAs that were examined. Moreover, 5S rRNA and GAPDH also had low rankings in the stability analysis during skin wound healing, which was consistent with the previous reports [38, 39]. Although U6 has been the most commonly used RG for miRNA quantification in skin disorder studies, the stability of U6 has not yet been evaluated and verified in wounded skin. This leaves questions about whether this normalization approach is accurate. To our knowledge, this was the first study to investigate the stability of U6 in the skin wound healing process. Our results using the four algorithms suggested that U6 had a low ranking amongst the RGs examined, indicating that U6 is not a proper internal normalizer for miRNA qPCR analysis.

In forensic practice, biomarkers used for wound age estimation are frequently influenced by post-mortem autolysis or putrefaction. Therefore, evaluating the vulnerability of miRNAs is of great forensic interest for skin samples experiencing post-mortem insults. The small size of miRNAs reportedly makes these molecules less susceptible to the degradation associated with chemicals and post-mortem autolysis or putrefaction [11, 40, 41]. In addition, miRNAs have been used as the RGs for numerous studies regarding PMI estimation using miR-122, miR-133a and 18S in heart tissue, and miR-133a, circ-AFF1 and LC-LRP6 in skeletal muscle tissue, indicative of prioritized stability for miRNAs [13, 14]. Similar to previous reports [15, 42, 43], our results demonstrated that the stabilities of most of the miRNA candidate RGs examined were superior to those of the comRGs, amongst which GAPDH and 18S had the lowest overall stability rankings. Therefore, GAPDH and 18S may be useful for PMI estimation, but not as internal normalizers. In addition, LC-Ogdh, a circular RNA (circRNA), was found to have low stability in the post-mortem skin samples tested in the present study. However, it was reported to be the most stable RG in post-mortem liver tissue samples in mice and used as an RG for studies on PMI estimation [13, 14]. For U6, no report was found on its stability in post-mortem skin tissue. Our data showed that the U6 Ct value increased significantly at a PMI of 4.5 days in the mouse skin, whereas others have shown it to be slightly raised at a 1.5-day PMI in rat spleen and at a 2-day PMI in mouse heart, liver, and skeletal muscle tissues at the same experimental conditions (temperature: (25±2)°C, humidity: (55±3)% [14, 42]. The increase in Ct values with a longer PMI might be associated with differences in species, organ, or degradation speed. However, consistent with previous studies, we found U6 to be more stable than 18S, GAPDH, and LC-Ogdh in the post-mortem skin samples.

Using a comprehensive analysis with the four aforementioned statistical algorithms, we validated mmu-miR-30d-5p, mmu-miR-152-3p, and mmu-miR-26a-5p as the prioritized RGs in stability for miRNA quantification in the wounded skin samples over various PTIs and PMIs. This was further verified using human samples. It has been accepted that the stability of a single internal control is not sufficient to accurately calibrate data, and the reasonable selection of multiple comRGs or miRNAs as RGs can thus improve the accuracy of data analysis [23, 32, 44]. Compared with the arithmetic mean of multiple RGs, the geometric mean can better control for possible outlying values and abundance differences between various genes, as well as more accurately average the RGs [23]. Therefore, we propose to use the geometric mean of a set of miRNAs in combination, such as miR-26a-5p, miR-30d-5p, and miR-152-3p, as an internal control for miRNA quantification in injured skin samples. However, no studies on the involvement of these three miRNAs in skin wound repair have been published. Evidence suggests that miR-26a-5p and miR-152-3p are stably expressed in a variety of cell or tissue types [45–53], reinforcing our supposition of their potential utility as stable RGs. There are currently no data showing that miR-26a-5p or miR-30d-5p expression levels are affected by any disease. Only a few studies have suggested that the expression of mmu-miR-152-3p is related to the pathogenesis of tumours: overexpression of mmu-miR-152-3p is related to hepatitis B virus related hepatocellular carcinoma and significantly suppress breast cancer cell proliferation, migration, and invasion [54, 55].

In recent years, numerous studies have been conducted on the roles of miRNAs in skin injuries [55–60]. For example, miR-142, as inflammation-related miRNAs, mice with miR-142 overexpression have significantly delayed the healing of *Staphylococcus aureus*-infected skin damage compared with wild-type mice [61, 62]. Neutrophils are the primary immune cells in the early inflammatory response during skin wound repair [63]. Neutrophil-derived miR-142 is required to promote neutrophil migration and increase the ability of the wound site to resist microbial infection [61]. Macrophages are another type of immune cell that govern the inflammatory phase during skin wound repair. Interestingly, miR-21 and miR-223 participate in the regulation of macrophage polarization in cutaneous wounds [64, 65], with miR-21 being critical in not only the inflammation phase, but also the proliferation phase of this process. Inhibition of miR-21 can delay the re-epithelialization process, with impaired wound contraction also being suppressed [66, 67]. Overexpressing miR-21, miR-29b, miR-106b, and miR-146a can reportedly accelerate re-epithelialization and reduce excessive scar generation in wound healing [62]. Contrary to this effect, downregulating miR-200c, miR-210, and miR-155 is effective in improving wound healing. Because these miRNAs play such unique roles in skin injury healing, it is reasonable to estimate skin wound age by evaluating changes in their expression levels. No study has thus far reported the use of miRNAs as markers for skin wound age estimation, suggesting that it is a completely new research direction and highlights the novelty of our work.

Our study does have some shortcomings. First, using a combination of miR-26a/30d/152 may make the experiment more tedious with more complex statistical analysis. However, this approach is necessary to avoid the effects of post-mortem degradation. Second, miRNAs can also degrade, but are still more stable than various proteins and other biological indicators. In the future, a more in-depth analysis of miRNA stability should be conducted.

In conclusion, our results demonstrate that utilizing a combination of miR-26a/30d/152 is recommended as a normalization strategy for miRNA quantitative analysis in wounded skin samples. Accordingly, investigations on skin wound age estimation can potentially be conducted using miRNAs as RGs, which can support accurate skin wound age estimation. Further research will be performed on this in the future.

## Authors’ contributions

Rui Zhao and Dawei Guan conceived and designed the research. Longlong Suo, Jian Cheng, and Haomiao Yuan wrote the main manuscript text and performed the lab experiments. Zhenfei Jiang, Dilichati Tash, Linlin Wang, and Hao Cheng performed the animal experiments. Zhongduo Zhang, Fuyuan Zhang, Miao Zhang, and Zhipeng Cao performed the statistical analysis. All authors contributed to and approved the final version of the manuscript.

## Compliance with ethical standards

The use of animals was approved by the Animal Experiment Committee of China Medical University (cmu2019119). All animal experiments were conducted according to the Guide for the Care and Use of Laboratory Animals and approved by the Animal Experiment Committee of China Medical University. All procedures performed in studies involving human samples were approved by the Scientific and Ethical Committee of China Medical University (ethical review [2018] 062) and in accordance with the 1964 Declaration of Helsinki and its later amendments. Written informed consent was obtained from all participants.

## Disclosure statement

None declared.

## Funding

This study was supported by the National Natural Science Foundation of China [grant numbers 81871529, 81971793, 81801874], National Key Research and Development Program of China [grant number 2018YFC0807204], and Liaoning Natural Science Foundation [grant number 20180550722].

## Supplementary Material

Supplementary_Material_owad037
